# Efficacy of infliximab in neuro-Behçet’s disease presenting with isolated longitudinally extensive transverse myelitis

**DOI:** 10.1007/s00415-013-7150-5

**Published:** 2013-11-01

**Authors:** Ryo Kuroda, Junko Suzuki, Mizuho Muramatsu, Asami Tasaki, Mariko Yano, Noboru Imai, Masahiro Serizawa, Masahiro Kobari

**Affiliations:** 1 Department of Neurology, Japanese Red Cross Shizuoka Hospital, 8-2 Ohte-machi, Aoi-ku, Shizuoka 420-0853 Japan; 2 Department of Neurology, National Hospital Organization Utano Hospital, Kyoto, Japan; 3 Departments of Rheumatology, Japanese Red Cross Shizuoka Hospital, Shizuoka, Japan

Dear Sirs,

Behçet’s disease (BD) is a relapsing systemic inflammatory disorder of unknown etiology, showing common characteristics of oral and genital ulcers and uveitis. Several prospective studies have shown that neurological complications of BD, generally called neuro-Behçet’s disease (NBD), occur in approximately 5–14 % of patients with BD [1–3]. In NBD, the frequency of spinal cord involvement is 8–14 % [4, 5].

Use of tumor necrosis factor (TNF)-alpha antagonist therapy has been described for NBD [6–8]. Nevertheless, reports of NBD with spinal cord involvement describing the efficacy of anti-TNF-alpha therapy are scarce [9]. We describe herein a case of NBD with longitudinal myelitis refractory to standard therapy that was successfully treated using infliximab, a TNF-alpha antagonist.

A 43-year-old Japanese man was admitted to our hospital with acute paralysis of both legs, and bowel and bladder incontinence. He had presented with headache at 33 years old. Recurrent oral ulceration, genital ulceration, and positive pathergy reaction were identified, and he had fulfilled the International Behçet’s disease Study Group criteria [10].

On admission, examination showed spastic paraparesis with hyperreflexia in the lower extremities, including pathological reflexes. Results from blood tests were unremarkable, and negative results were obtained for both HLA-B51 and anti-aquaporin-4 antibody. Spinal magnetic resonance imaging (MRI) showed abnormally bright signals on T2-weighted imaging in the T12 spinal cord. Cerebrospinal fluid (CSF) analysis demonstrated increased lymphocytes (23 cells/mm^3^) and protein (78 mg/dl). CSF interleukin (IL)-6 was markedly elevated (214 pg/ml, normal <4.0 pg/ml). CSF IgG oligoclonal banding was negative. We diagnosed NBD with isolated extensively transverse myelitis. Treatment with intravenous methylprednisolone (m-PSL) at 1,000 mg/day for 3 days improved symptoms. A maintenance dose of 15 mg/day of oral PSL was administered.

Seven months later, his gait gradually deteriorated and he became paraparetic. Intravenous m-PSL pulse therapy was administered, but his disease remained active. A persistent high CSF IL-6 concentration (60.2 pg/ml), which exceeded the critical threshold (> 20 pg/ml) for progression of neurological manifestations in chronic progressive NBD [11], indicated that his clinical course had proceeded to the secondary progressive type. The patient was administered oral methotrexate (6–10 mg/week) for 4 months, but little improvement was seen and his walk remained slow and spastic.

**Fig. 1:**
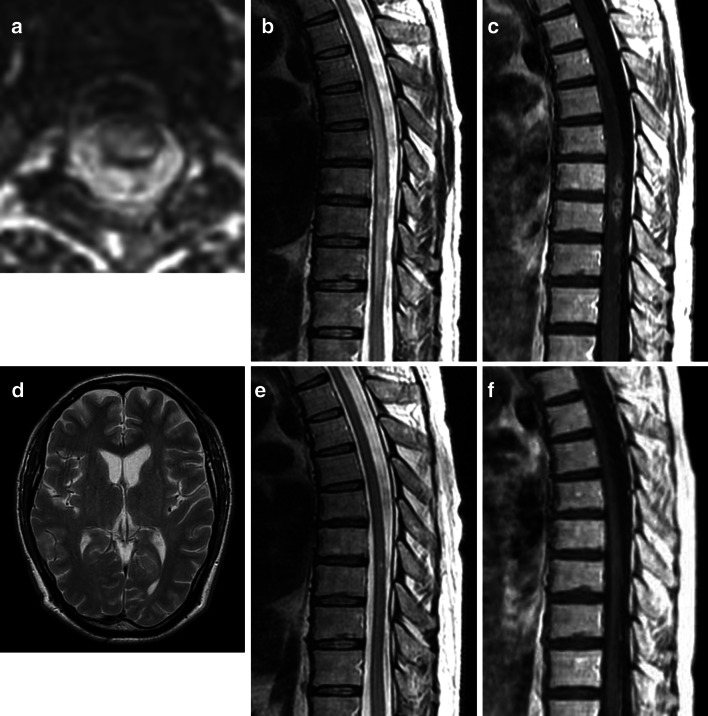
Spine magnetic resonance imaging (MRI) before (**a**–**c**) and after (**e, f**) infliximab therapy and cranial MRI (**d**). **a** Axial T2-weighted image showing transverse myelitis. **b** Sagittal T2-weighted image showing abnormally increased longitudinal T2 signal with mild enlargement from T6 to T10. **c** Sagittal contrast-enhanced T1-weighted image revealing partial enhancement of the lesion. **d** Cranial MRI had no abnormal finding. **e** Sagittal T2-weighted image showing reduced longitudinal abnormal signal. **f** Sagittal contrast-enhanced T1-weighted image revealing no enhancement lesion

Twelve months after the first admission, he suddenly became totally paraplegic and was rehospitalized. Sensations of pinprick, temperature, and vibration were totally absent in both legs. MRI of the thoracic spine showed an abnormally increased longitudinal T2 signal from T6 to T10 (Fig. 1a, b). Contrast-enhanced T1-weighted imaging revealed partial enhancement of the lesion (Fig. 1c). Cranial MRI had no abnormal finding (Fig. 1d). The CSF level of IL-6 was extremely high (424 pg/ml). Despite receiving IV m-PSL pulse therapy, no clinical improvement was obtained, and he could neither bend his knees nor transfer to a wheelchair by himself. Intermittent urethral catheterization was needed due to severe urinary dysfunction.

Infliximab was started (5 mg/kg at weeks 0, 2, and 6, and every 8 weeks thereafter). A dramatic improvement in his severe spasticity was noticed within 24 h after initiating the first infusion and remained stable throughout the observation period. Repeat MRI of the spine performed 2 weeks later revealed reduction of longitudinal abnormal signals on T2-weighted imaging (Fig. 1e). The lesions with contrast enhancement disappeared (Fig. 1f). CSF IL-6 was reduced to 18.3 pg/ml. At 4 weeks, the patient was able to stand unaided, and use of intermittent catheterization was stopped. Over the subsequent 6 months, he has maintained clinical improvement (detailed clinical course in Table 1), and has achieved the ability to walk while holding tables. No adverse effects were seen during the observation period.

**Table 1:**
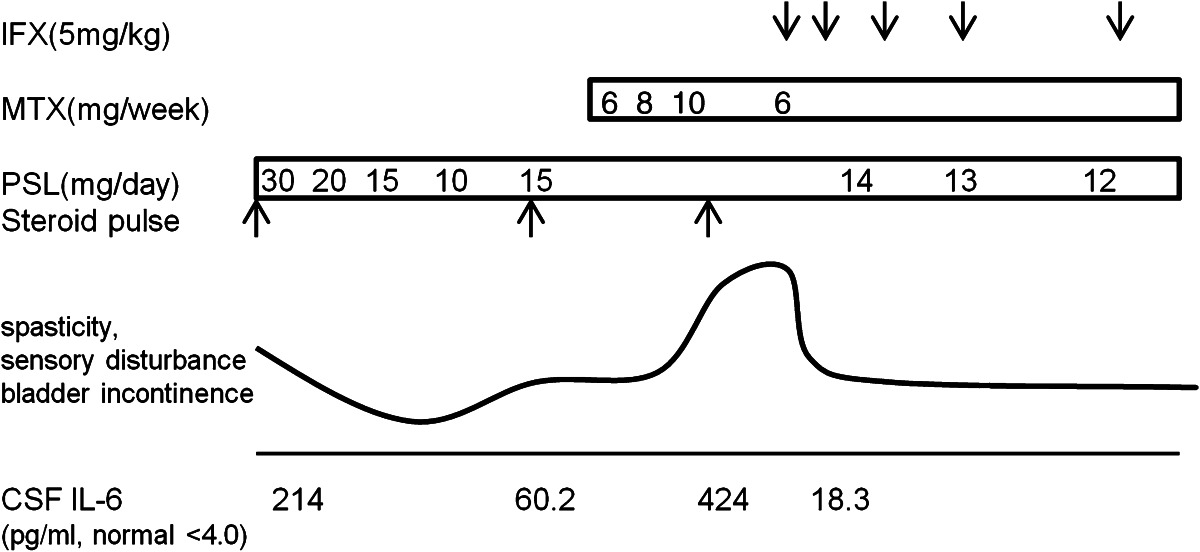
Clinical course of symptoms and CSF findings and the treatment regimen

*PSL* prednisolone, *MTX* methotrexate, *IFX* infliximab, *CSF* cerebrospinal fluid, *IL-6* interleukin-6

We have confirmed the efficacy of infliximab in a refractory NBD patient with isolated spinal cord involvement. A previous study suggested that NBD patients with spinal cord involvement show worse prognosis than those with other types of NBD [12]. In that report, over 70 % of the 24 patients showed a primary or secondary progressive course, and eight patients died, despite administration of steroid and immunosuppressants. Appropriate therapeutic intervention is thus needed to prevent the development of irreversible neurological sequelae.

Inflammation in BD is thought to be mediated by cytokines, particularly TNF-alpha, which is located upstream of cytokine networks, including the pathway that produces IL-6 [13]. Some articles on TNF-alpha antagonist therapy for steroid/immunosuppressant-refractory NBD involving other neurological sites have been published, and most have reported satisfactory clinical responses to therapy without severe side effects [6–8]. However, only one report has described NBD with spinal cord involvement that was successfully treated using anti-TNF-alpha therapy [9].

To the best of our knowledge, this represents the first published report of NBD with spinal cord involvement successfully treated using infliximab according to a detailed cytokine profile. We confirmed reduction in CSF levels of IL-6 after infliximab treatment, along with diminished disease activity. This finding supports previous reports about other clinical phenotypes of NBD [13, 14].

No consensus has been reached regarding the optimal timing to start infliximab therapy. The present case showed a number of poor prognostic factors, including spinal cord involvement, multiple attacks, progressive course, and high CSF levels of IL-6. We suggest earlier initiation of infliximab therapy in the presence of abnormal CSF IL-6 concentrations and other poor prognostic factors.

In conclusion, this case supports the view that infliximab is a potent therapeutic option for refractory NBD cases with spinal cord involvement.
